# Misalignment-Tolerant Planar Spiral Coil Pair Design for 13.56 MHz Inductive Coupling of Wireless Resistive Analog Passive Sensors

**DOI:** 10.3390/s24030752

**Published:** 2024-01-24

**Authors:** Babak Noroozi, Bashir I. Morshed

**Affiliations:** 1 Electrical and Computer Engineering Department, FAMU-FSU College of Engineering, Tallahassee, FL 32310, USA; 2 Department of Computer Science, E. E. Whitacre Jr. College of Engineering, Lubbock, TX 79409, USA; bmorshed@ttu.edu

**Keywords:** biomedical monitoring, distance measurement, genetic algorithm, inductive coupling, optimization, planar spiral coil, wearables, wireless sensors

## Abstract

Long-term daily-life body signal monitoring offers numerous advantages, such as timely response to health alerts, diseases monitoring, and reducing time and expenses related to clinical trials. Access to physiological data can be achieved with low-cost and comfortable wireless wearable sensors. In our previous publication, we reported a low-cost, easy to implement, and unobtrusive wireless resistive analog passive (WRAP) sensor to provide a feasible bio-signal monitoring technique by using a pair of printed spiral coils (PSC) in a near field connection. Sensitivity, defined as the response to the transducer, is a critical feature in the establishment of a reliable system. In the previous publication, we presented the utilization of a Genetic Algorithm to design a pair of coils and related components to maximize sensitivity. Although the coils’ misalignment can significantly affect the optimized sensitivity, it was not incorporated into the optimization process. This paper focuses on optimizing the coils and components in order to maximize both their sensitivity and their resilience against movements of the PSC pair. In a square-shaped pair comprising a primary coil of 60 mm and a secondary coil of 20 mm dimensions, we found that the sensitivity is maximized at 1.3 mƱ for a 16 mm axial distance. Additionally, it remains above 0.65 mƱ within ±11.25 mm lateral and +14 mm axial displacements.

## 1. Introduction

Continuous monitoring of bio-signals over a long-term period provides a reliable approach for the early detection of disease and tracking the severity of biomarkers in a patient. It also eliminates the costly presence of patients in hospitals for a short-time sampling of vital signals. A lightweight wearable sensor that is easy to carry, with no interference in daily activities, is the key feature necessary to achieve this idea. Our proposed wireless and passive sensor [1] eliminates obtrusive wires and the need for a power supply, exhibiting characteristics such as being lightweight, low-cost, and maintenance-free. These are attainable through the magnetic inductive connection between a primary and secondary printed spiral coil (PSC). In a previous study [1], the coupling factor (mutual inductance) was assumed as a constant value, yet in practice, it varies based on the coils’ specifications and their relative position. This paper incorporates the coils’ size and their relative position into the optimization process to maximize the sensitivity and minimize their susceptibility to misalignment.

Bluetooth [2], WiFi [3], near field communication (NFC) [4], and near-field RFID [5] are other wireless techniques used to transfer bio-signals. While an active circuit in WiFi and Bluetooth-based connection enhances communication distance and SNR, these solutions tend to be costly, heavy, reliant on a power supply, and, consequently, less suitable for long-term wearable applications. The NFC and near-field RFID methods also employ an inductive magnetic connection between two coils, but they require an active chip on the sensor side, leading to a requirement for power transfer or harvesting that results in complicated and costly circuits. In contrast, our proposed simple and low-cost Wireless Resistive Analog Passive (WRAP) sensor offers a simple concept enabling their fabrication on a flexible and disposable substrate [6]. A varactor sensor can also be used to sense the bio-signals [7,8] instead of our resistive transducer approach in the inductive wireless coils. A capacitive sensor modulates the frequency in response to a bio-signal, adding complexity to the receiver circuit, and requires a wide bandwidth due to its frequency shift. In addition, these sensors are susceptible to the influence of stray capacitors and, since the varactor directly affects the resonance frequency, the coils’ profiles must be redesigned for any specific sensor. In contrast, a resistive sensor output can be detected by a simple amplitude demodulator with a narrower frequency band, and the coils’ profile and resonance circuits are not affected by the sensor varieties. The coil profiles play a key role in maximizing the sensitivity of the WRAP sensor. The research on coil optimization is limited to wireless power transfer (WPT), NFC, and RFID applications. In a WPT application, the coils are optimized for maximizing the power transfer efficiency as the objective function [9,10,11]. The coil optimization study on NFC and RFID techniques focuses on impedance matching and metal proximity [12,13]. We proposed our novel approach, utilizing a Genetic Algorithm, to design a pair of coils for maximum sensitivity ([1,14]). The variable relative position of untied coils in a real setting degrades the optimized sensitivity through shifting the coupling factor of the coils. In this paper, the coil profile is optimized for maximum sensitivity along with minimum susceptibility to coil misalignments.

In our simplified coil optimization [1], we assumed a constant value for the coupling factor and, consequently, the coil distance.

In this study, the mutual inductance variation has been incorporated to the coil optimization process. The goal was to optimize the coil and the circuit components for maximum sensitivity and robustness against changes in the coil’s position. Research on compensating for the coils misalignments is also a focus in the field of Wireless Power Transfer (WPT). While the extra coils or loops are employed to compensate the coils displacement in some studies [15,16], other compensation methods are application-specific [17,18]. In [18] a turn distribution algorithm is proposed to uniform the primary magnetic field. This method requires a complex and case-specific design and additionally, it reduces the magnetic field in the coaxial area of the coils to achieve uniformity on the sides. In [19] the effect of misalignment on power efficiency is minimized by adjusting the components to maximize the power efficiency function relative displacement. In our novel approach, we incorporate coils’ misalignment by integrating the coupling factor into the optimization process. In this model, the sensitivity is maximized at a practical coupling factor value that minimizes the susceptibility to misalignment. The theoretical results are verified by FEA simulation in COMSOL Multiphysics. 

The remainder of this paper is organized as follows. The circuit schematic, coil models, and equations are introduced in Section 2. The optimization method, objective function, constraints, and boundaries are described in Section 3. The measurement setup is explained in Section 4 and the results are compared with simulation and analytical results. Section 5 discusses the results and analyzes the effect of components and fabrication tolerances on the results. The paper is closed with a clear conclusion in Section 6. 

## 2. Model and Equations

**Figure 1:**
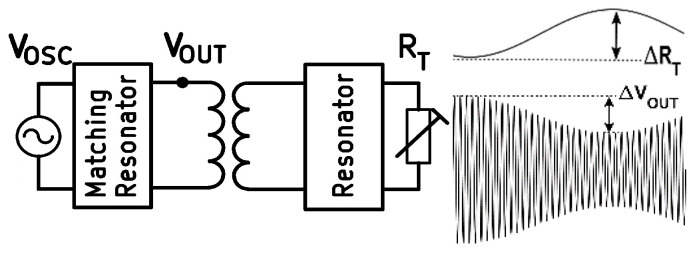
(**left**) System overview. (**right**) Sensitivity is defined as Δ*V_Out_* in response to Δ*R_T_*.

**Figure 2:**
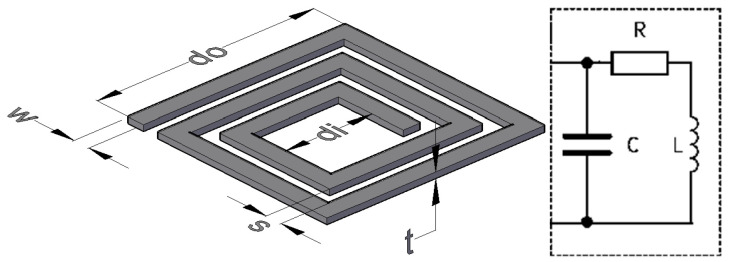
(**left**) Printed Spiral Coil, (**right**) the equivalent circuit.

Figure 1 shows the concept of the proposed WRAP sensor. *R_T_* represents the transducer resistor, which converts a bio-signal to a variable resistance. The passive secondary circuit affects the inductive magnetic field generated by the primary circuit where the variable transducer resistance modulates the primary coil’s voltage, as is illustrated in Figure 1. Figure 2 shows a PSC with the physical specification and equivalent circuit. The Current Sheet equation [20] from four other expressions has been adopted to calculate the self-inductance as it has previously shown the best match with the experimental results [1].

(1) $$L=\frac{1.27\mu_{0}\left(d_{O}+d_{i}\right)n^{2}}{4}\left[ln\left(\frac{2.07}{\varphi }\right)+0.18\varphi +0.13\varphi^{2}\right]$$

where $\mu_{0}$ is the air magnetic permeability, *d_O_* and *d_i_* are shown in Figure 2, and φ (fill-ratio) is defined in (2):

(2) $$\varphi =\frac{d_{O}-d_{i}}{d_{O}+d_{i}}=\frac{d_{O}-\left[d_{O}-2nw-2\left(n-1\right)s\right]}{\;d_{O}+\left[d_{O}-2nw-2\left(n-1\right)s\right]}=\frac{n\left(s+w\right)-s}{\;d_{O}-n\left(s+w\right)+s}\;$$

*s* and *w* are specified in Figure 2, and *n* is the number of turns. The PSC’s resistance (3), including the skin and proximity effects, has been embraced from [21] due to its best match with the experimental results.

(3) $$R\approx R_{DC}\left[1+\frac{d^{2}.f\pi \mu_{0}\sigma }{4.48}\right]$$

*R_DC_* is the DC resistance that is defined by (4), *d* is the diameter of circular cross-section wire with the equivalent area to the rectangular conductor on the PCB with track width and thickness of *w* and *t*, respectively, and is defined by (5), *f* is the frequency, and *σ* is the track’s conductivity in siemens Ω^−1^.

(4) $$R_{DC}=\frac{L_{C}}{t.W.\sigma }$$

(5) $$d=\sqrt{\frac{tw}{\pi }}$$

*L_C_* is the conductor’s length and for a PSC with *n* turns, conductor’s width (space) *w* (*s*), and the outer size *d_O_* (Figure 2) is calculated by (6).

(6) $$L_{C}=4nd_{O}-3nw-{\left(2n-1\right)}^{2}\left(s+w\right)$$

**Figure 3:**
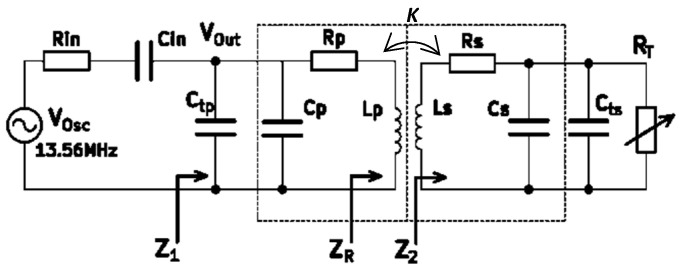
Circuit schematic of WRAP system.

The complete circuit schematic is shown in Figure 3. The circuit is derived using a 13.56 MHz (ISM radio band) signal generator with internal resistor *R_in_*. The signal generator and the rest of the circuit are matched through a capacitor *C_in_*. The two external capacitors, *C_tp_* and *C_ts_* along with *L_P_* and *L_S_*, tune the resonance frequency on 13.56 MHz. The sensitivity is the circuit responses to the transducer’s change and its normalized value is defined in (7).

(7) $$Sensitivity=\frac{d\left(V_{Out}/V_{Osc}\right)}{dR_{Transducer}}\;({Ω}^{-1})$$

To formulize the sensitivity in detail, according to Figure 3, the following equations can be derived:

(8) Z2RT=RS+jωLS+11RT+jωC2

where *C*_2_ = *C_ts_* + *C_S_*

(9) $$Z_{R}\left(R_{T}\right)=\frac{{\left[M\times \omega \right]}^{2}}{Z_{2}\left(R_{T}\right)}=\frac{{\left[k\times \omega \right]}^{2}L_{P}L_{S}}{Z_{2}\left(R_{T}\right)}\;(\mathrm{Reflected Impedance})$$

(10) $$M=k\sqrt{L_{P}L_{S}}\;(\mathrm{Mutual Inductance and Coupling factor})$$

(11) $$Z_{1}\left(R_{T}\right)=1/\left(j\omega C_{1}+\frac{1}{R_{P}+j\omega L_{P}+Z_{R}\left(R_{T}\right)}\right)\;(C_{1}\;=\;C_{tp}\;+\;C_{P})$$

(12) $$Z_{in}=R_{in}+\frac{1}{j\omega C_{in}}$$

(13) $$\frac{V_{OUT}\left(R_{T}\right)}{V_{in}}=\frac{Z_{1}\left(R_{T}\right)}{Z_{1}\left(R_{T}\right)+Z_{in}}$$

(14) $$\mathrm{Sensitivity}\;\left(R_{T}\right)=\frac{d\left(V_{OUT}/V_{OSC}\right)}{dR_{T}}=\frac{d}{dR_{T}}\left(\frac{Z_{1}\left(R_{T}\right)}{Z_{1}\left(R_{T}\right)+Z_{in}}\right)$$

## 3. Optimization

### 3.1. Genetic Algorithm (GA)

The sensitivity as the multivariable objective function for the PSC design can be expressed in the following equation:

(15) $$\mathrm{Sensitivity}=f\left(R_{T},C_{in},C_{1},C_{2},k,\;n_{1},s_{1},\;w_{1},\;n_{2},s_{2},\;w_{2}\right)$$

In this study, the transducer resistance is assumed to be 1 KΩ. While this assumption does not impact the generality of the study, the effect of various transducer resistances is discussed in Section 5. As a result, sensitivity becomes a function of ten variables and, due to its non-linearity and complexity, the Genetic Algorithm (GA) has been employed for optimization; it has previously been proven to be an appropriate method to address such a problem [1]. In this paper, our optimization approach has been improved in two aspects compared to the previous study. First, the two-steps used for the optimization of components and coil specifications have been combined into a single step, and, second, the coupling factor (*k*) has been introduced as a new variable in the objective function. In a GA optimization, the variables must be bounded by upper and lower limits, and the problem may have some constraints that need to be defined. The coupling factor (*k*) requires special attention when defining the boundaries. Theoretically, the coupling factor (*k*) varies from 0 and 1, depending on the coupling between the primary and secondary coils that indicates the portion of flux from one coil that passes through the other coil. If the upper limit for *k* is defined as 1, the optimal coupling factor is determined to be more than 0.1, while, in practice within our physical settings, the coupling factor is less than 0.1. Therefore, we set the upper limit for the coupling factor as 0.1, which represents the maximum achievable *k* in our setup. As the optimal value for the coupling factor is consistently found near the upper bound, the lower bound value is not critical, and it is set at 0.06 to maintain a margin between the two bounds. According to the chosen PCB fabrication facility (Oshpark LLC, https://oshpark.com/), the minimum values for *s* and *w* are 0.152 mm (6 mil), imposing boundaries on the track’s width (*w*) and the tracks’ space (*s*) (Figure 2). In addition to the boundaries, two constraints defined by (16) and (17) must be added to the optimization process.

(16) $$d_{i}>0\overset{\;}{\Rightarrow }d_{O}-2nw-2\left(n-1\right)s>0\;\left(i=1,2\right)$$

(17) $$\varphi_{1}\geq 0.85$$

The first constraint (16) ensures the feasibility of the coil structure for both primary (*i* = 1) and secondary (*i* = 2) coils, where the optimum *n*, *s*, and *w* values maintain *d_i_* (Figure 2) as a non-zero value. The second constraint (17) keeps the fill-factor of the primary coil high enough to guarantee a uniform flux density at the center of the coil, which is discussed in Section 5 (Discussion). The lower and upper boundaries of the variables are listed in Table 1. The capacitor ranges in Table 1 are experimentally determined to narrow down the domain of the variables in the optimization algorithm. They need to be redefined for a new setting. Table 2 shows the applied setting in this optimization problem.

### 3.2. Susceptibility to the K Variation

The coupling factor indicates the portion of the generated magnetic flux by one coil that intersects the other coil. The relative positions of the primary and secondary coils have a significant effect on the coupling factor. The dependency of sensitivity on coupling factor (*k*) is analytically provided by (8)–(14). However, the relation between the coils’ alignment and the coupling factor is not theoretically well-defined. Several expressions have been suggested in the literature [22,23,24] for calculating the mutual inductance between two PSCs with different positioning; however, as previously explained [1], they are not well-aligned with the experimental results. Therefore, we employ the Finite Element Analysis (FEA) simulation approach to determine the correlation between the coupling factor and the position of the coils. The accuracy of this approach has been validated in the previous report [14]. In summary, the dependency of sensitivity on coil position is derived by integrating the sensitivity-coupling factor analytical equation with the simulation curve for the coupling factor and coil position. Hence, we minimize the effect of misalignment on sensitivity through minimizing the effect of coupling factor variation on sensitivity and the effect of coil movement on coupling factor.

### 3.3. Minimizing the Dependency of Sensitivity on Coupling Factor Variation

**Figure 4:**
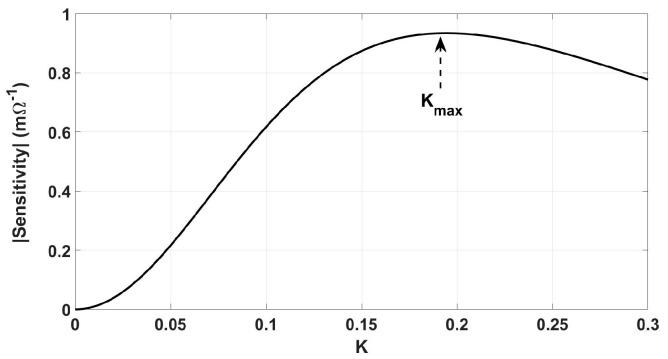
A typical sensitivity-*k* curve.

Figure 4 shows a typical sensitivity-coupling factor curve that is derived from (8)–(14). If *k_O_* is the optimum coupling factor, to minimize the dependence of sensitivity on *k*, *k_O_* must be equal to *k_max_* in Figure 4. According to the setting of this study, including the size of the primary and secondary coils and their distance, the maximum achievable coupling factor is *k_max_* ≤ 0.1. Due to the stochastic nature of the Genetic Algorithm, the optimization results are not necessarily unique in several runs and since the *k_max_* is defined as the upper boundaries in Table 1, *k_O_* ≈ *k_max_* is more likely within the results. Table 3 shows the *k*-sorted results for multiple runs and *d*_*O*1_ = 60 mm and *d*_*O*2_ = 20 mm. In a trade-off between maximum sensitivity and coil distance (the smaller *k_O_* corresponds to further distance) the first row in Table 3 with minimum *k_O_* (maximum coil distance) is picked as the optimum profile.

### 3.4. Minimizing the Dependency of Coupling Factor on Coils’ Alignment

**Figure 5:**
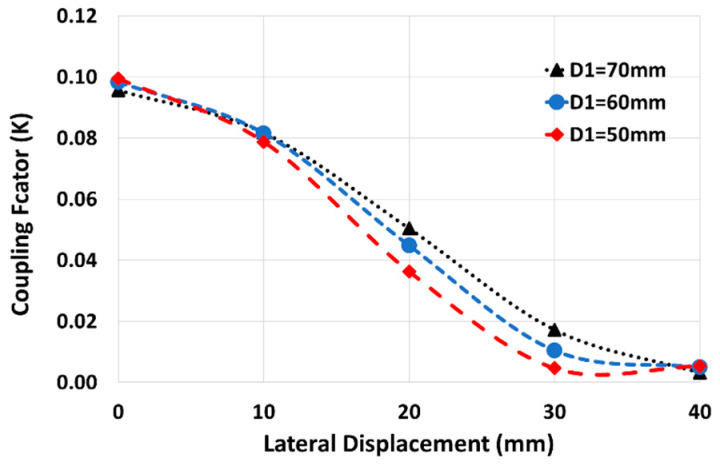
The effect of primary size on the coupling factor with lateral displacement (Φ ≈ 0.9).

**Figure 6:**
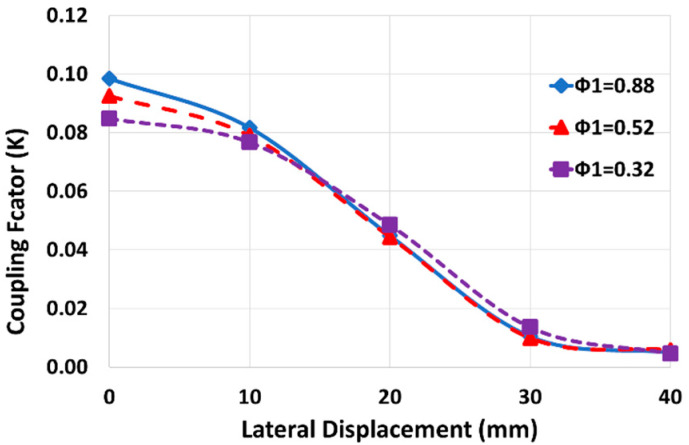
The effect of primary fill-factor on the coupling factor (D_1_ = 60 mm, Distance = 16 mm).

The coupling factor between two printed spiral coils depends on several factors, including coil size, the magnetic permeability of the surroundings, and the coils’ fill-factors (2). According to the untied coils in our wearable application setup, the gap between coils is filled by air. For user comfort, the secondary coil size is set as small as 20 mm, thus, the adjustable parameters for coil shape are restricted to the primary and secondary fill-factor and the primary size. As the secondary fill-factor has a negligible impact on the coupling factor; our focus is on minimizing the dependency of the coupling factor on the coil’s relative position by optimizing the primary coil size and its fill-factor. We showed in [14] that the coupling factor and its uniformity (with coils’ axial/lateral distance) increases with the primary coil size, as shown in Figure 5. In addition, the coil resistance increases by primary size, which leads to a lower sensitivity. Therefore, the larger equivalent resistance in a larger primary coil decreases the sensitivity and, on the other hand, it slightly uniforms the coupling factor in lateral displacements. Considering the area occupied by the primary coil and according to Figure 5 (and results in [14]), 60 mm is taken as the optimal selection for the primary size, particularly for lateral distance less than 30 mm. Figure 6 shows the effect of the primary fill-factor ($\varphi_{1}$) on the coupling factor and its variation with lateral displacement for a primary size of 60 mm and an axial distance of 16 mm. According to this figure, the larger fill-factor increases the coupling factor, especially at coaxial distances, and it leads us to set a constraint, as shown in (17).

## 4. Fabrication, Simulation, and Measurement Results

**Figure 7:**
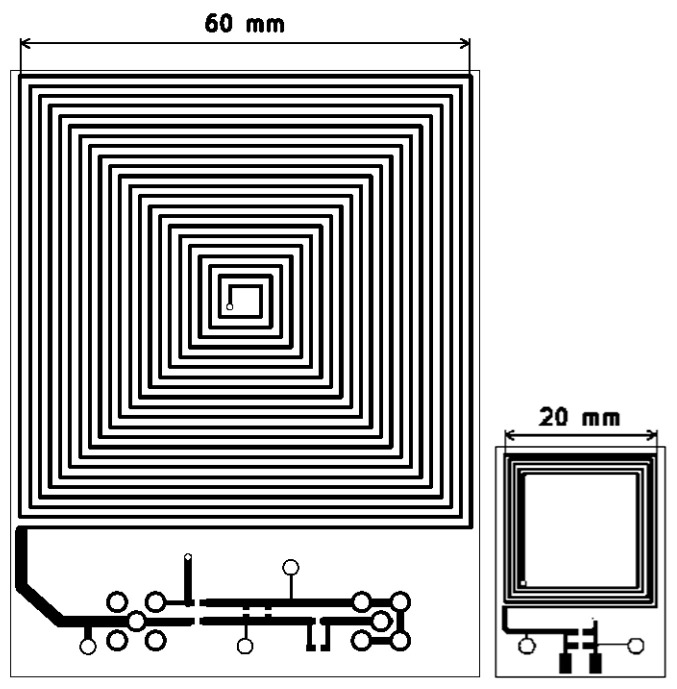
PCB design for primary (**left**) and secondary (**right**).

**Figure 8:**
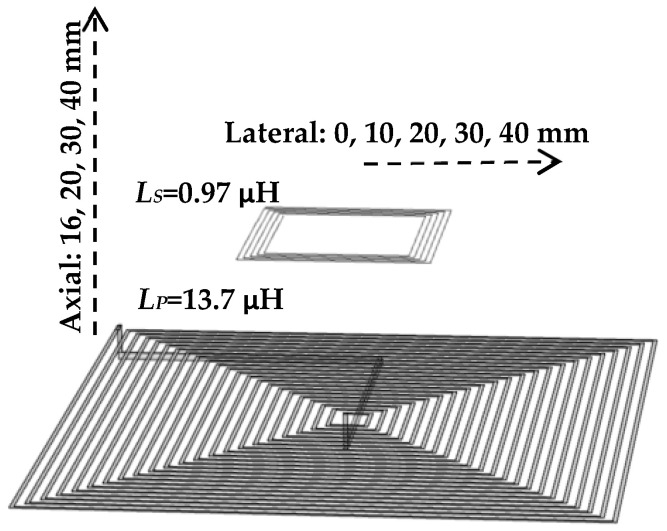
The axial distance/lateral distance between primary and secondary in the sensitivity simulation and measurement.

The first row in Table 3 shows the optimum coil pair with the maximum sensitivity, corresponding to the minimum *k_O_* and the boundaries/settings in Table 1 and Table 2 and the constraints in (16) and (17). Figure 7 shows the designed PCB with KiCad (version: 5.1.5-3, KiCad EDA). Table 4 shows the measured equivalent components for the primary and the secondary coils with an LCR Analyzer (Agilent 4294A, 40 Hz–110 MHz, Agilent Technologies, Santa Clara, CA, USA). The coupling factor and sensitivity were measured for the coil’s relative arrangements, as illustrated by Figure 8.

### 4.1. Coupling Factor

We utilized the technique recommended by [25] for measuring the coupling factor. In this method, the self-inductance of the series connection of the primary and secondary coils are measured using an LCR analyzer for both in-phase (*L_I-P_*) and opposing-phase (*L_O-P_*) connections. Through (18), the mutual inductance can be calculated by using the two measured self-inductances. Finally, (10) is applied to determine the coupling factor.

(18) $$M=\frac{L_{I-P}-L_{O-P}}{4}$$

**Figure 9:**
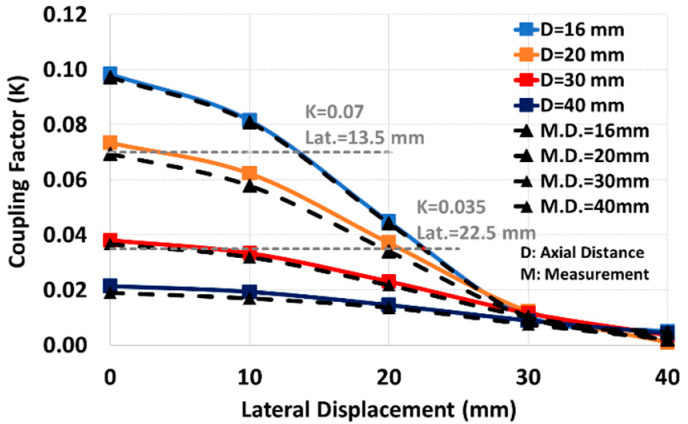
Experimental and simulation results of coupling factor for optimum coil in different lateral and axial distances.

We used COMSOL (COMSOL Inc., Burlington, MA, USA) as an FEA tool to simulate the coils’ mutual and self-inductance. The coupling factor experimental and simulation results are in good agreement as they are shown and compared in Figure 9.

### 4.2. Sensitivity

**Figure 10:**
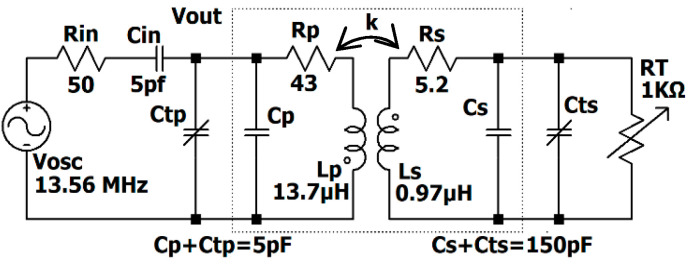
The final schematic.

**Figure 11:**
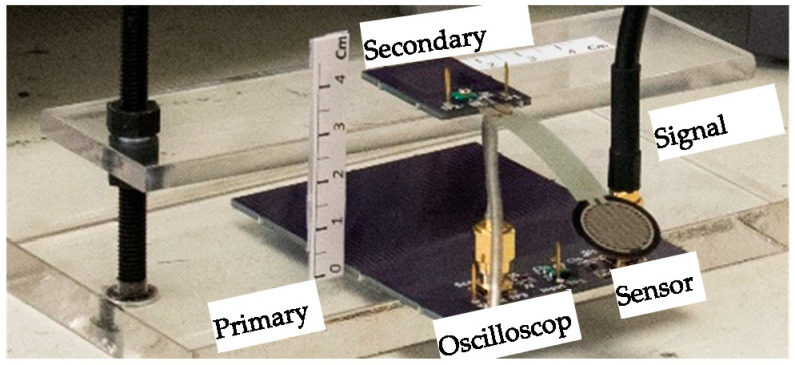
The sensitivity measurement setup.

**Figure 12:**
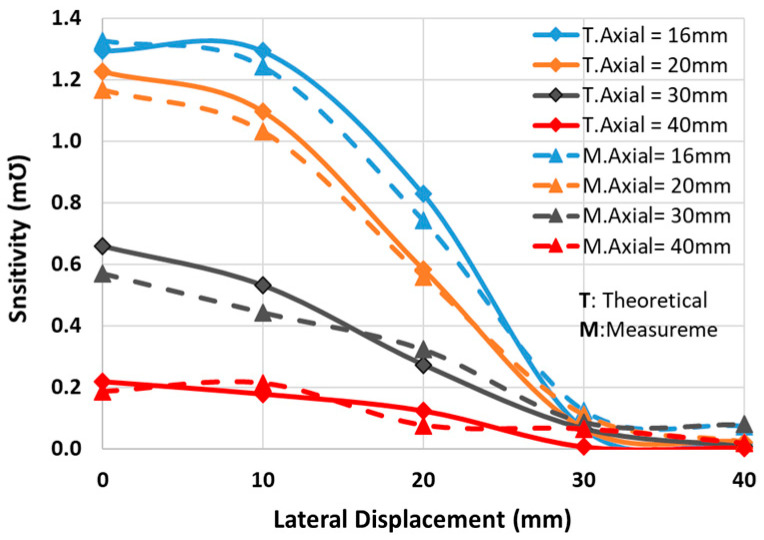
The measured and calculated sensitivity vs lateral displacements for different axial distances.

**Figure 13:**
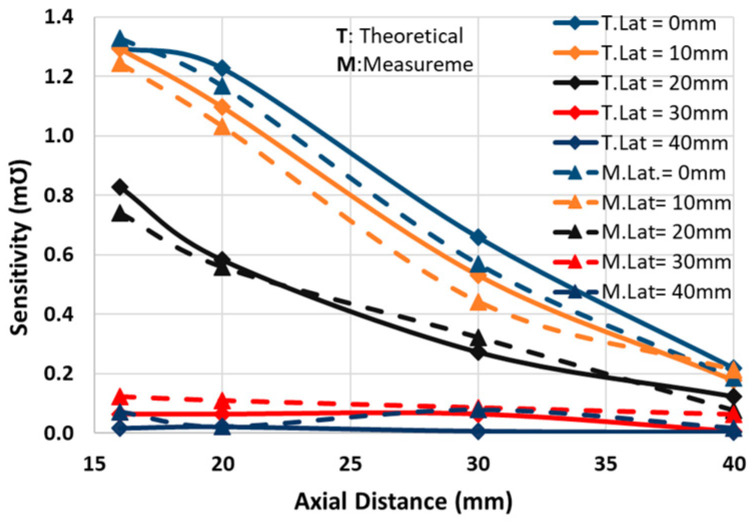
The measured and calculated sensitivity vs axial distances for different lateral displacements.

The final schematic with the coils’ measured equivalent values is shown in Figure 10. The optimum capacitors are fine-tuned by the trimmers in primary and secondary circuits (*C_tp_* and *C_sp_*). According to the measured values in Figure 10, the analytical sensitivity has shifted from 1.29 mƱ in Table 3 to 1.32 mƱ, indicating a difference of less than 2.5%. The setup depicted in Figure 11 is used to measure the sensitivity for the positions of the coils shown in Figure 8. Theoretical sensitivity is calculated by the LTspice (Linear Technology, Milpitas, CA, USA) simulation tool where the axial and lateral distances of the coils are correlated with the coupling factor driven from the measured data in Figure 9. To measure the practical sensitivity, *R_T_* was swept from 0.850 kΩ to 1.1 kΩ and the *V_out_* was measured with an oscilloscope (Agilent, Model DSO-X 2024A, Tektronix Probe TPP0200, Agilent, Santa Clara, CA, USA). The primary and secondary capacitors were fine-tuned by individually connecting the primary and secondary circuits to a sweep signal generator and comparing the frequency and voltage measurement and simulation results. The loading effect of an oscilloscope probe with a 12 pF equivalent capacitor was almost attenuated by an 18 kΩ resistor in series with the probe. Figure 12 and Figure 13 show the sensitivity experimental and simulation results for the different axial and lateral displacements defined in Figure 8. According to these figures, for axial/lateral distances less than 20 mm, the measurements show a deviation of less than 5% from the theoretical results. The small *V_out_*, primarily due to attenuation, is a significant factor contributing to this discrepancy.

## 5. Discussion

**Figure 14:**
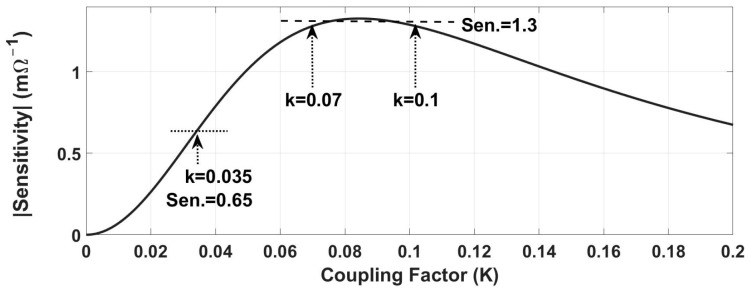
The sensitivity with coupling factor for the final schematic.

**Figure 15:**
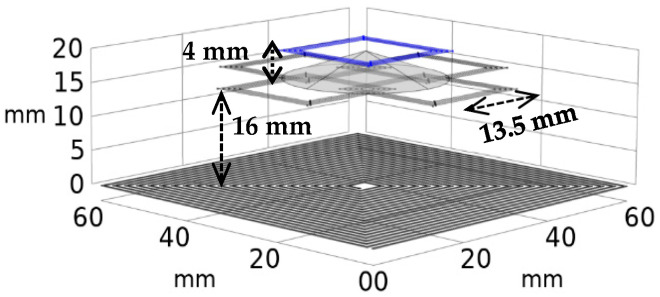
The secondary lateral and axial movement region in which the sensitivity is unchanged (Sen. ≈ 1.3 mƱ, 0.07 ≤ k ≤ 0.1).

**Figure 16:**
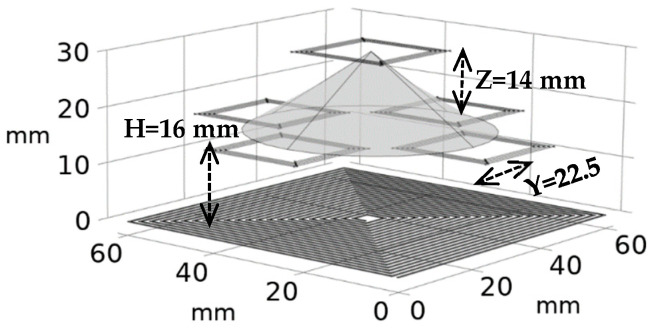
Read-Zone: the region in which the secondary can move, and the minimum sensitivity is more than half of its maximum (0.65 ≤ Sen. ≤ 1.3).

The effect of misalignment on the sensitivity was minimized by minimizing the dependency of sensitivity on the coupling factor (Figure 4) and of the coupling factor on axial/lateral displacements (Figure 5 and Figure 6). Figure 14 shows how the coupling factor affects the sensitivity for the circuit in Figure 10. According to Figure 14, when *k* = 0.1, the sensitivity remains almost unaffected by axial/lateral misalignment, as long as 0.07 ≤ *k*. Considering Figure 9, the range of coupling factor (*k*) corresponds to the secondary misalignment range shown in Figure 15. As illustrated in Figure 15, when the center of the secondary coil moves within an imaginary cone, with a base positioned 16 mm above the primary coil, a height of 4 mm, and a based radius of 13.5 mm, the sensitivity remains relatively constant. Figure 16 introduces another hypothetical cone wherein the sensitivity does not decrease beyond half of its maximum value while the center of the secondary coil moves inside, which is denoted as the Read-Zone hereafter. Figure 15 and Figure 16 are the results of integrating Figure 9 and Figure 14 for measurement results.

The Read-Zone size shown in Figure 16 can be affected by the components and PCB fabrication tolerance. The PCB tolerances are defined by the PCB fabrication service (Oshpark LLC) as follows:

$$\Delta s=\pm 25.4\;\mu m\;\left(\pm 1\;\mathrm{mil}\right),\;\Delta w=\pm 12.7\;\mu m\;\left(\pm 0.5\;\mathrm{mil}\right)$$

Due to the insignificant effect of PCB tolerances on the coupling factor, we only analyze the PCB tolerances on the sensitivity-coupling factor equation. In this equation, the parameters influenced by the PCB tolerances are the equivalent resistance and self-inductance of the coils. The peak and the slope of the sensitivity-coupling factor (*k*) curve (Figure 14) are affected by PCB tolerances, resulting in corresponding changes to the Read-Zone (Figure 16). There are 2^4^ possible changes in the primary and secondary optimal profiles (±∆*s*_1_, ±∆*w*_1_, ±∆*s*_2_, ±∆*w*_2_). The sensitivity-coupling factor (*k*) curve can be analyzed for various possible PCB fabrication errors (Figure 14). Table 5 lists the worst-case PCB error which has the most effect on sensitivity. According to this table, the sensitivity is reduced from 1.3 mƱ to o.8 mƱ by PCB error, leading to a smaller Read-Zone, as specified in Table 6. Additionally, the Read-Zone (Figure 16) is affected by the capacitors’ tolerances (±∆*C*_1_, ±∆*C*_2_, and ±∆*C_in_*). The simulation results indicate that *C_in_* is the component with maximum effect on the sensitivity where its ±5% tolerance causes a 10% decrease in sensitivity. The effect of different error sources and their associated sensitivity and Read-Zone are summarized in Table 6. According to this table, while the fabrication tolerance decreases the height and side of the Read-Zone by almost 3 mm, the capacitor’s tolerance does not significantly change this region.

**Figure 17:**
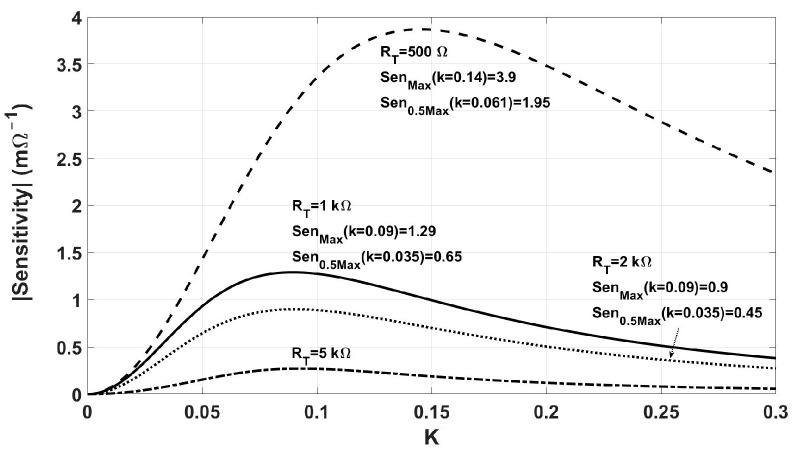
The effect of *R_T_* on sensitivity. The smaller RT increases the sensitivity; however, it increases the *k_max_*.

The effect of *R_in_* and *R_T_*. Although *R_in_* and *R_T_* are fixed at 50 Ω and 1 kΩ, respectively, their values may change in different circuits and transducers. The effect of *R_in_* and *R_T_* on the Read-Zone are analyzed through their effect on the sensitivity-coupling factor (*k*) curve (Figure 14), while the changes in the coupling factor are neglected. The combination of Figure 9 with the updated sensitivity-coupling factor (*k*) curve determines the new Read-Zone. Figure 17 shows the effect of *R_T_* on the sensitivity-coupling factor (*k*) curve. According to this figure, the maximum sensitivity and the Read-Zone are reduced by larger *R_T_*. On the other hand, a 50% decrease in *R_T_* (from 1 kΩ to 500 Ω) triples the sensitivity, increasing it from 1.3 mƱ to 3.9 mƱ; however, it requires a coupling factor of 0.14, which is too large to achieve at a distance greater than 10 mm. The effect of *R_T_* on the maximum sensitivity and Read-Zone is summarized in Table 7.

**Figure 18:**
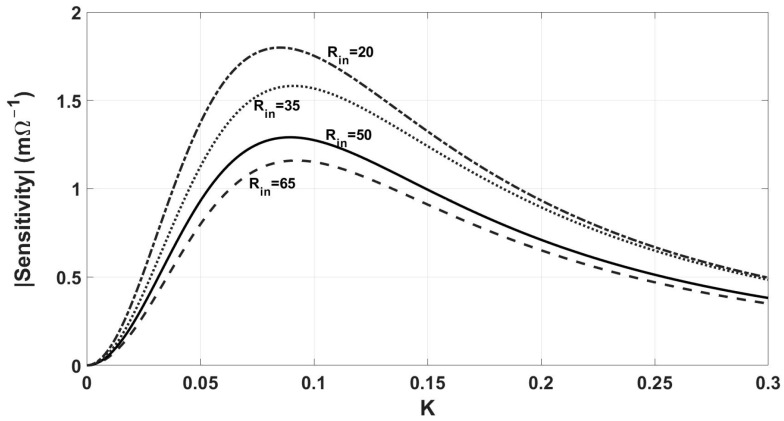
The effect of *R_in_* on the sensitivity and *k_max_*. The sensitivity and *k_max_* decrease with *R_in_*.

Figure 18 shows the effect of *R_in_* on the sensitivity-coupling factor (*k*) curve. Regarding this figure, while the maximum sensitivity increases with lower *R_in_*, as indicated in Table 8, the Read-Zone shows little variation within the range of 20 Ω to 65 Ω for *R_in_*.

## 6. Conclusions

The 13.56 MHz signal generated by the scanner is modulated by the resistive sensor at the secondary side through the near field magnetic connection between the primary and secondary coils. The electrical components and the coils’ profiles determine the maximum sensitivity and the Read-Zone, the secondary span region where the sensitivity remains greater than half of its maximum value. In this study, we used a Genetic Algorithm to optimize the coil profile and the components in order to maximize the sensitivity and the Read-Zone within fabrication and application constraints. To minimize the effect of coil misalignment on sensitivity, we first minimized the sensitivity to a coupling factor (*k*) variation and then minimized the effect of the coils’ relative position on the coupling factor (*k*) by finding the appropriate coil size and fill-factor. A pair of optimized coils were fabricated for the primary and secondary with sizes of 60 mm and 20 mm, respectively. The measured coupling factor over different primary-secondary alignments was verified by FEA simulation. In addition, the analytical and experimental results for sensitivity in various alignments exhibited a close match, showing a difference of less than 5% in the region within 20 mm lateral and axial misalignments. We showed that while the fabrication tolerances can reduce the sensitivity to 40% of its maximum value, they do not change the Read-Zone significantly. Furthermore, we showed that while the tolerance of the impedance matching capacitor (*Cin*) is the component with the most influence on sensitivity, its 5% tolerance results in a 15% decrease in sensitivity, yet it does not affect the Read-Zone significantly. We also analyzed the effect of the transducer and the signal generator internal resistors on sensitivity and the Read-Zone. We found that a transducer and a signal generator resistor in the range of 1~2 kΩ and 20~65 Ω, respectively, do not considerably change the Read-Zone. The smaller transducer resistance, however, can drastically increase the sensitivity. Significant increase in sensitivity is achievable by smaller transducer resistance; however, this would lead to a notable increase in susceptibility to alignments unless the coupling factor were to increase accordingly. Moreover, the sensitivity is increased moderately by decreasing the signal generator internal resistor (*R_in_*). The evaluation of different possible ways for improving the coupling factor, such as multilayer PCB, can be future directions of study in order to expand the sensitivity and its Read-Zone.

## Figures and Tables

**Table 1:** The lower and upper boundaries of variables in GA.

Bound	*R_T_* (kΩ)	*C_in_* (pF)	*C*_1_ (pF)	*C*_2_ (pF)	*k*	*n*	*s* (mm)	*w* (mm)
Lower	1	5	5	50	0.06	3	0.152	0.152
Upper	1	20	20	200	0.1	30	70	100

**Table 2:** GA option settings.

**Population Initialization**	Size	3000
**Stopping Criteria**	Max Stall Generation	50
	Max St. Time	Inf.
	Max. gen.	200
**Fitness scaling**		Rank
**Selection Function**		Stochastic uniform
**Mutation**	Function	Adaptive Feasible
**Crossover**	Fraction	0.3
	Function	Scattered
**Elite**	Elite count	150 (5% Pop.)

**Table 3:** GA multiple runs for the upper/lower boundaries in Table 1 and: *d_O_*_1_ = 60 mm, *d_O_*_2_ = 20 mm.

*R_T_* (kΩ)	*C_in_* (pF)	*C*_1_ (pF)	*n*_1_	*s*_1_ (mm)	*w*_1_ (mm)	*R_P_* (Ω)	*L_P_* (µH)	$\varphi_{1}$	*C*_2_ (pF)	*n*_2_	*s*_2_ (mm)	*w*_2_ (mm)	*R_S_* (Ω)	*L_S_* (µH)	$\varphi_{2}$	*k_O_*	*Sen*(*k_O_*) (mƱ)
1	5	5	22	0.91	0.41	45.85	13.68	0.88	150	5	0.30	0.30	5.77	0.89	0.16	0.090	1.29 *
1	5	5	23	0.64	0.66	45.31	13.93	0.95	140	6	0.61	0.15	6.99	1.00	0.25	0.090	1.27
1	5	5	22	0.15	1.12	44.62	14.02	0.86	143	7	0.76	0.15	7.30	0.98	0.39	0.092	1.22
1	5	5	22	0.79	0.51	45.88	14.10	0.86	139	5	0.33	0.15	6.51	1.04	0.12	0.094	1.30
1	5	5	22	0.74	0.58	44.73	13.51	0.89	150	4	0.15	0.15	5.50	0.87	0.06	0.094	1.34
1	5	5	23	0.86	0.43	46.58	14.15	0.93	150	5	0.41	0.15	6.39	0.97	0.14	0.094	1.20
1	5	5	22	0.43	0.86	44.48	13.75	0.88	100	6	0.33	0.15	7.60	1.34	0.15	0.096	1.65
1	7	5	20	0.15	1.24	40.50	11.59	0.86	145	5	0.36	0.20	6.12	0.96	0.14	0.096	1.28
1	5	5	22	0.15	1.12	44.62	14.02	0.86	92	6	0.20	0.15	7.88	1.55	0.11	0.098	1.72
1	5	5	23	0.99	0.33	46.76	13.71	0.96	104	5	0.15	0.15	6.77	1.25	0.07	0.098	1.60
1	20	5	14	1.70	0.43	29.03	5.53	0.88	82	6	0.15	0.15	7.99	1.65	0.09	0.100	0.89
1	5	5	22	0.15	1.14	43.84	13.49	0.90	74	6	0.15	0.15	7.99	1.65	0.09	0.100	1.91
1	5	5	22	1.07	0.25	47.88	13.83	0.87	50	9	0.23	0.15	10.99	2.63	0.19	0.100	1.94
1	5	5	22	0.33	0.97	44.23	13.66	0.88	50	9	0.15	0.23	10.36	2.59	0.20	0.100	2.08

*: The maximum sensitivity with minimum *k.*

**Table 4:** The fabricated coils’ components and their calculated values (Table 3).

	Primary		Secondary	
	*L* (µH)	*R* (Ω)	*L* (µH)	*R* (Ω)
Calculation	13.68	45.85	0.89	5.77
Measurement	13.7	43	0.97	5.2
Error	0.14%	6.2%	9%	9.9%

**Table 5:** The worst-case fabrication tolerances (+∆*s*_1_, +∆*w*_1_).

Coil Spec.	Primary				Sensitivity (mƱ)		
	*s* (mm)	*w* (mm)	*L* (µH)	*R* (Ω)	*k* = 0.09	*k* = 0.035	*k* = 0.045
Optimum	0.91	0.41	13.7	45.8	1.3	0.6	0.9
Worst-case	0.94	0.42	12.9	44.6	0.8	0.3	0.4

**Table 6:** The maximum sensitivity and the Read-Zone for the worst-case PCB and capacitor tolerances.

	The Read-Zone				
	*H* (mm)	*Z* (mm)	*Y* (mm)	*k*	Sen. (mƱ)
Optimum	16	14	22.5	0.035–0.1	0.65–1.3
Fabrication Error (Worst case)	16	11	20	0.045–0.1	0.4–0.8
*C_in_* (±5%)	16	14	22	0.038–0.1	0.5–1.1

**Table 7:** The effect of *R_T_* on the sensitivity and Read-Zone.

	The Read-Zone				
*R_T_* (kΩ)	*H* (mm)	*Z* (mm)	*Y* (mm)	*k*	Sen. (mƱ)
0.5	<10	<12	20	0.061–0.14	1.95–3.9
1	16	14	22.5	0.035–0.1	0.65–1.3
2	16	14	22.5	0.035–0.1	0.45–0.9

**Table 8:** The effect of *R_in_* on Read-Zone.

	The Read-Zone				
*R_in_* (Ω)	*H* (mm)	*Z* (mm)	*Y* (mm)	*k*	Sen (mƱ)
20	~16	14	22.5	0.035–0.085	0.9–1.8
35	16	~13	22	0.038–0.088	0.8–1.6
50	16	14	22.5	0.035–0.09	0.65–1.3
65	~16	~13	21.5	0.039–0.092	0.58–1.16

## Data Availability

Data are contained within the article.
